# CocoaMFDB: A dataset of cocoa pod maturity and families in an uncontrolled environment in Côte d'Ivoire

**DOI:** 10.1016/j.dib.2023.109196

**Published:** 2023-05-03

**Authors:** Kacoutchy Jean Ayikpa, Diarra Mamadou, Abou Bakary Ballo, Konan Yao, Pierre Gouton, Kablan Jérôme Adou

**Affiliations:** a ImVia, Université Bourgogne Franche-Comté, Dijon, France; b LaMI, Université Felix Houphouët-Boigny, Abidjan, Côte d'Ivoire; c UREN, Université Virtuelle de Côte d'ivoire, Abidjan, Côte d'Ivoire

**Keywords:** Data collection in agriculture, Smart agriculture, Images of cocoa pods, Ripe, Unripe, Data acquisition, Image preprocessing, Space color

## Abstract

Cocoa cultivation is the basis for chocolate production; it has a unique aroma that makes it useful in the production of snacks and usable for cooking or baking. The maximum harvest period of cocoa is normally once or twice a year and spread over several months, depending on the country. Determining the best harvesting period for cocoa pods plays a major role in the export process and the pods quality. The degree of ripening of the pods affects the quality of the resulting beans. Also, unripe pods do not have enough sugar and may prevent proper bean fermentation. As for too-mature pods, they are usually dry, and their beans may germinate inside the pods, or they may develop a fungal disease and cannot be used. Computer-based determination of the ripeness of cocoa pods throughout image analysis could facilitate massive cocoa ripeness detection. Recent technological advances in computing power, communication systems, and machine learning techniques provide opportunities for agricultural engineering and computer scientists to meet the demands of the manual. The need for diverse and representative sets of pod images is essential for developing and testing automatic cocoa pod maturity detection systems. In this perspective, we collected images of cocoa pods to set up a database of cocoa pods of the Côte d'Ivoire named CocoaMFDB. We performed a pre-processing step using the CLAHE algorithm to improve the quality of the images since the effect of the light was not controlled on our data set. CocoaMFDB allows the characterization of cocoa pods according to their maturity level and provides information on the pod family for each image. Our dataset comprises three large families, namely Amelonado, Angoleta, and Guiana, grouped into two maturity categories: the ripe and unripe pods. It is, therefore, perfect for developing and evaluating image analysis algorithms for future research.

**Specifications Table**

**Table:** 

Subject	Agricultural Sciences
Specific subject area	Monitoring of the maturity of cocoa pods and detection of the pod family
Type of data	Image XML File
How the data were acquired	The data was acquired by taking pictures with Nikon D500 and infinix cameras of cocoa pods from the plantation in the village of Yakassé 1. The images taken were stored in a computer
Data format	Analyzed Filtered
Description of data collection	The implementation of our cocoa pod dataset was done in three steps. First, the stage of photographing the pods was carried out on two different dates, namely May 26, 2022, and August 18, 2022. Then a classification of the images of cocoa pods was carried out according to the varieties of cocoa families and by categories of pods, namely ripe and unripe pods. Finally, we labeled the acquired images to locate all the cocoa pods present on each image and to proceed to the annotation of the identified pods.
Data source location	The images of cocoa pods are from the plantations of Yakassé 1, a village of Grand Bassam first capital of Côte d'Ivoire with a Latitude and longitude of 5°12′42″ north, 3°44′19″.
Data accessibility	Repository name: Mendeley Data Data identification number:10.17632/9msjjh3np6.2 Direct URL to data:https://data.mendeley.com/datasets/9msjjh3np6/2

## Value of the Data

• Images of cocoa pods obtained will be used in identifying cocoa types and varieties.
• These data are valuable to research activities whose aim is to monitor the link between a cocoa pod and beans.
• These data are a baseline for visualization under machine learning to get features in cocoa pod families identification and recognition.
• Harvests optimization is going to be practicable with a classification of the state of cocoa pods maturity.
• The segmentation of cocoa pods would allow the automatic location of cocoa pods in a given environment.

## 1 Objective

Côte d'Ivoire is the first cocoa producer in the world, and cocoa farming represents 14% of its GDP. In order to have better quality cocoa beans, it is necessary to shape the best cocoa pods harvesting period with regard to the state of ripe. Thus, unripe pods do not have enough sugar for a proper beans fermentation. As for overripe cocoa pods, dryness, and germination generally lead beans to development of fungal infections and unitability. This dataset would allow the set-up of automatic systems for harvest optimization.

### 1.1 Data Description

Cocoa pods were photographed with a Nikon D500 and Infinix cameras in a cocoa plantation located in Yakassé 1 village a few km from Grand Bassam in Côte d'Ivoire. The shots of these images were taken at different times of the day, in the morning between 9:30 a.m. and 11 a.m., and in the evening between 2 p.m. and 4 p.m., from different angles in an uncontrolled environment. The images of the pods were classified by category and family. The categories used are ripe cocoa pods and unripe cocoa pods. For each category, we obtained three main families which are: Amelonado, Angoleta, and Guiana.

• Amelonado

Amelonado comes from the Spanish word for melon-shaped. The fruits look like elongated melons, usually with a slight bottleneck. They have thick, usually smooth shells, rarely with some warts. The fruit has shallow grooves and a rounded tip. The Amelonado is a type of Theobroma cacao [1].

**Fig. 1:**
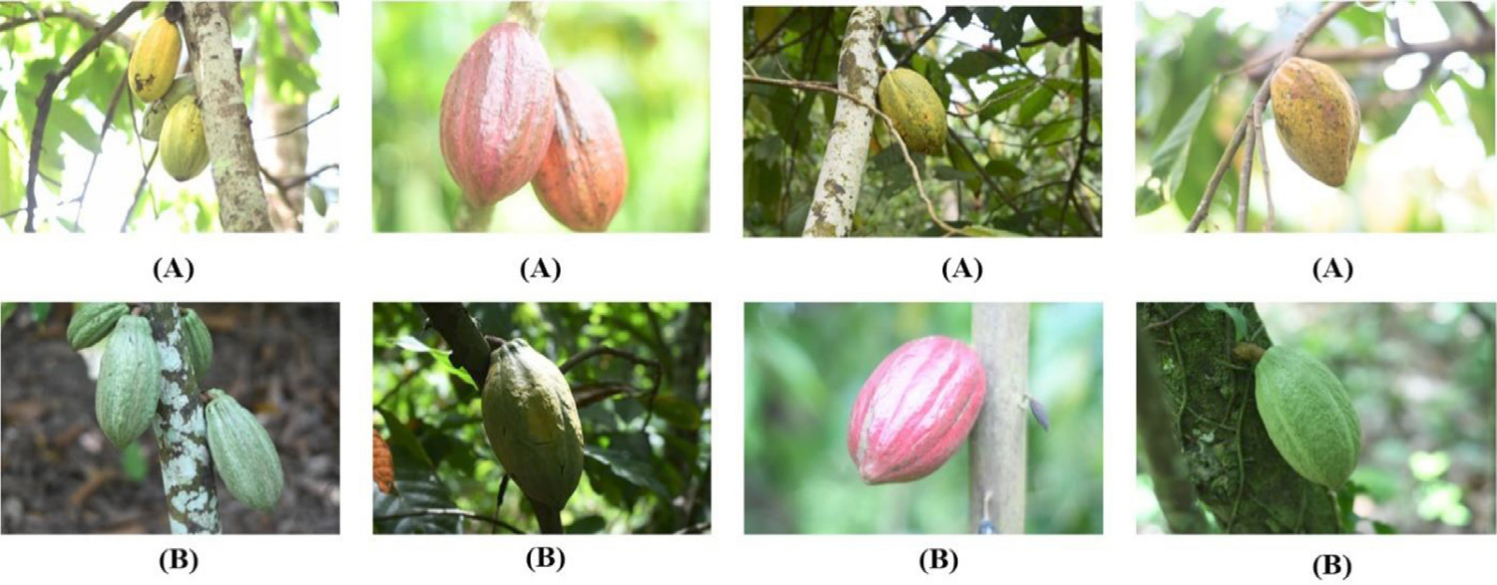
Images of Amelonado pods (A): Ripe Amelonado pods; (B): Unripe Amelonado pods.

Fig. 1 *shows the images of Amelonado cocoa arranged in two lines. The first line contains images of ripe Amelonado pods, and the second one contains unripe Amelonado pods.*

• Angoleta

The cocoa Angoleta is a derivative of the trinitario cocoa. It is the result of the crossing between criollo and forastero. Its shape is almost identical to that of a criollo, its surface is very rough, without bottleneck, it is a large fruit, with round seeds, and grooves in the endosperm of light purple color and has a superior quality [2].

**Fig. 2:**
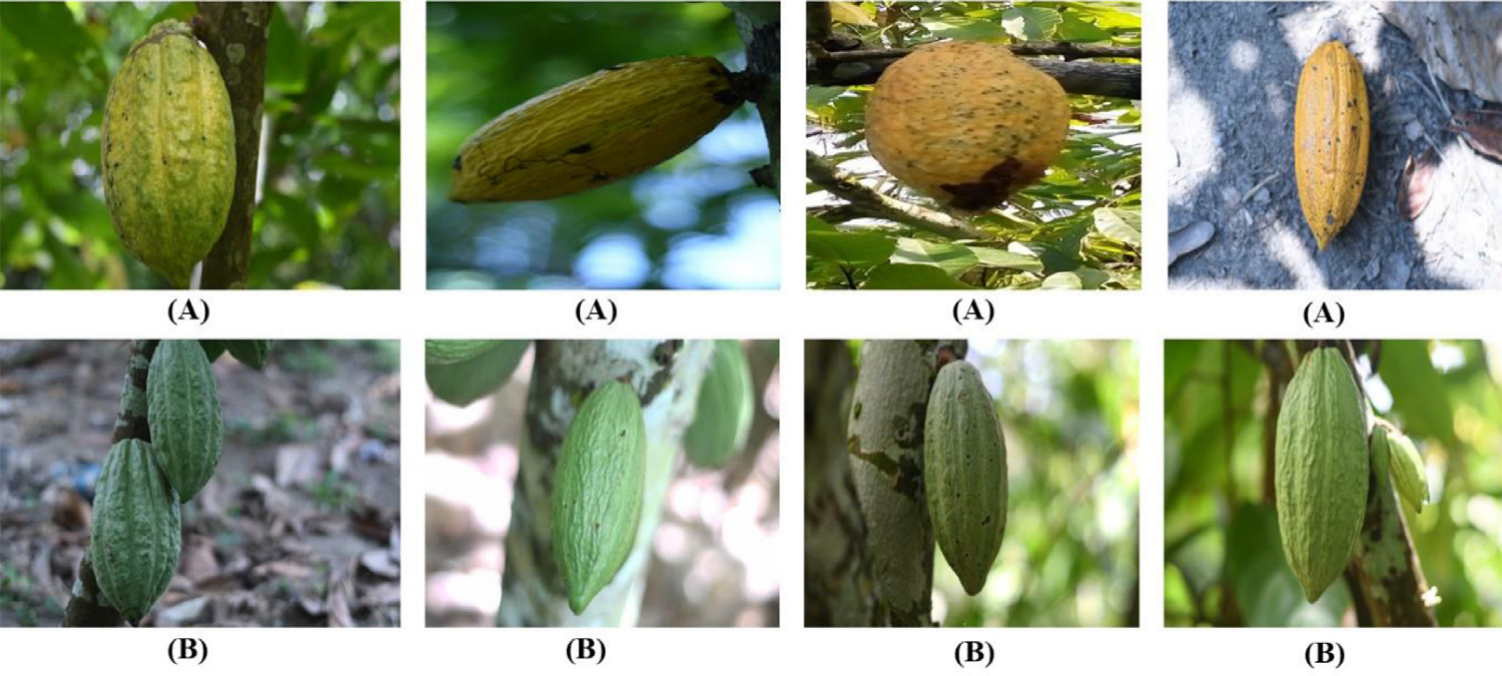
Images of Angoleta pods (A): Ripe Angoleta pods; (B): Unripe Angoleta pods.

Fig. 2 shows the images of Angoleta cocoa pods which are arranged in two lines. The first line contains the images of mature Angoleta pods and the second line the unmatured Angoleta pods.

• Guiana

Guiana is a little-known and endemic cocoa variety from French Guiana. Guiana cocoa differs from other cocoa trees by its characteristics such as marked furrows and verrucosity of its surfaces [3].

**Fig. 3:**
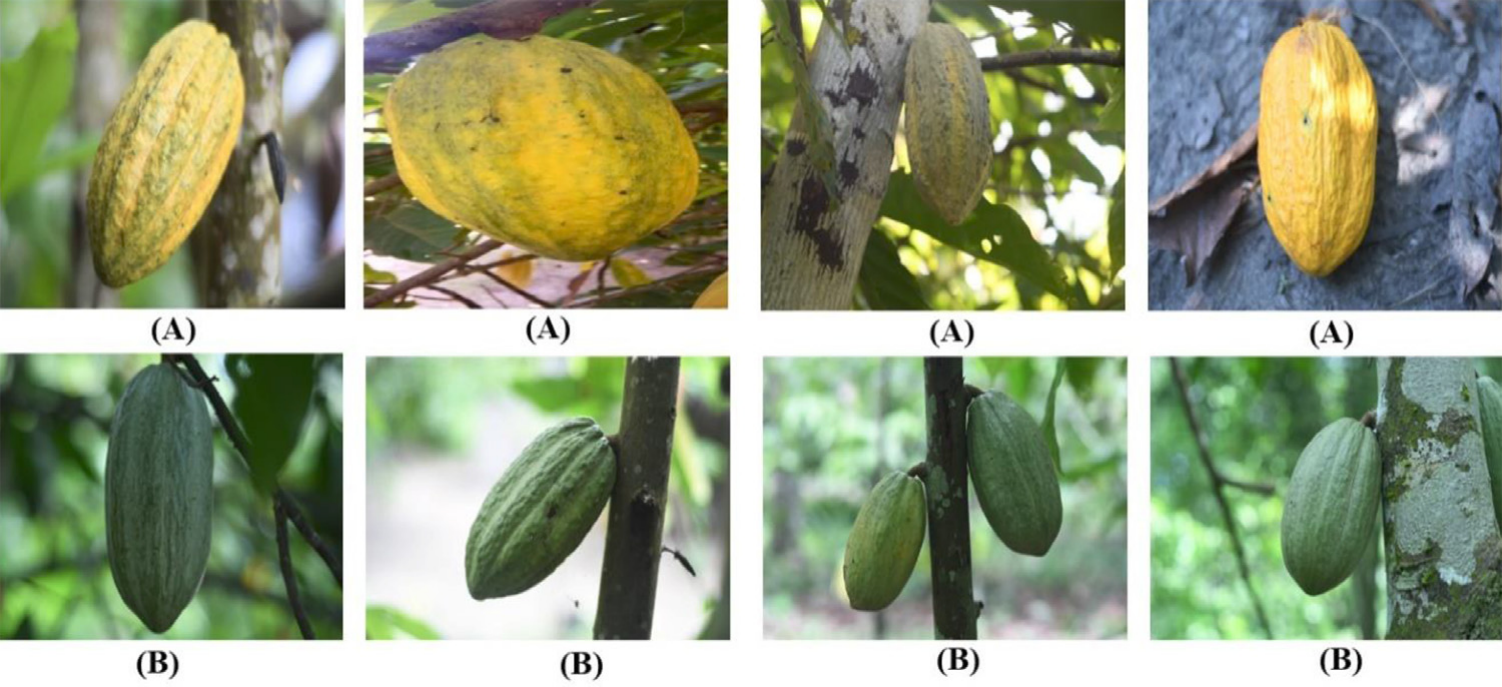
Images of Guiana pods (A): Ripe Guiana pods; (B): Unripe Guiana pods.

Fig. 3 *shows the images of Guiana cocoa pods which are arranged in two lines. The first line displays images of ripe Guiana pods and the second line contains images of unripe Guiana pods.*

• Data directory structure

**Fig. 4:**
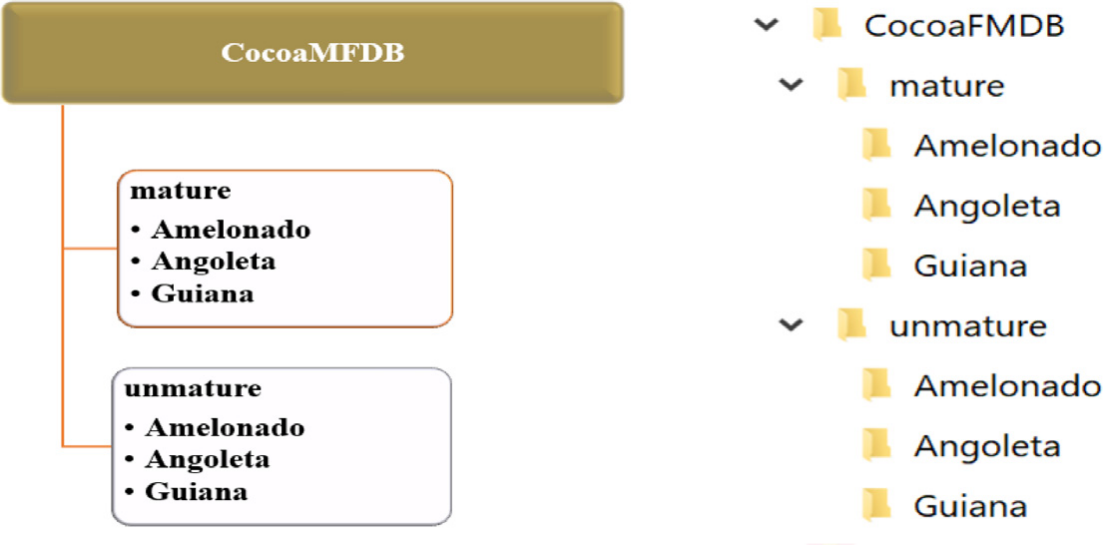
Structure of the dataset.

The directory to the dataset is structured as follows:

A parent directory named CocoaMFDB, then in the CocoaMFDB directory, two sub-directories named mature(ripe) and unmature(unripe) were set. Finally, each folder hosted the Amelonado, Angoleta, and Guiana directories. Fig. 4 shows the directory structure of the dataset

• Data summary

Table 1 *presents a summary of the cocoa pods images according to their type and maturity status.*

**Table 1:** Cocoa pod statistics: a brief summary.

Type	Ripe	Unripe	Total
Amelonado	260	245	**505**
Angoleta	170	133	**303**
Guiana	182	264	**446**
Total	**612**	**642**	**1254**

**Fig. 5:**
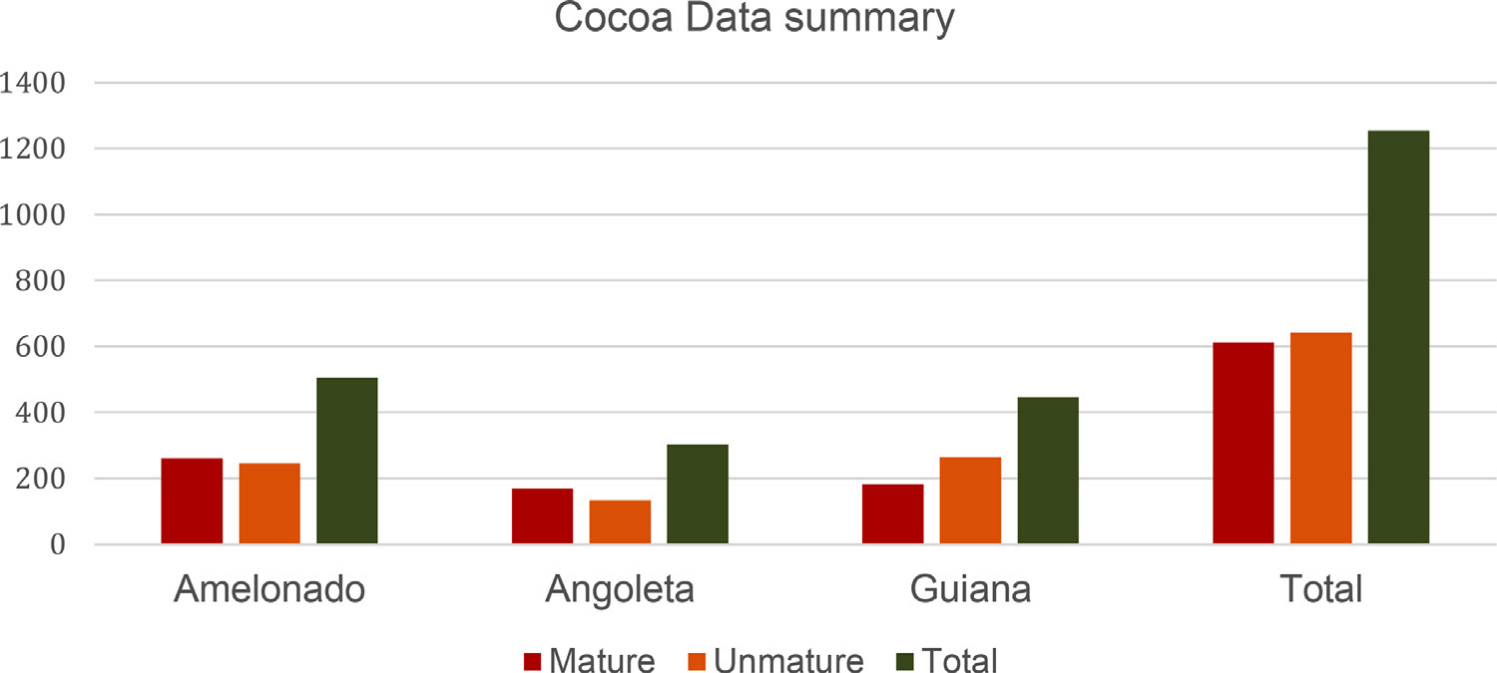
Histogram of the cocoa data summary.

Fig. 5*displays the histogram from to*Table 1.

## 2 Experimental Design, Materials and Methods

• Cocoa pods collection

Images collection took place in Yakassé 1 Grand Bassam on the following dates: May 26, 2022, and August 18, 2022. The images were captured from different angles in an uncontrolled environment, including various obstacles such as varying weather conditions such as wind, rain, fog, sun, etc., as well as changing lighting conditions such as the brightness, color, and direction of light, with the presence of shadows and reflections. The images contain occluded stems and leaves and cocoa pods.

• Data pre-processing

**Fig. 6:**
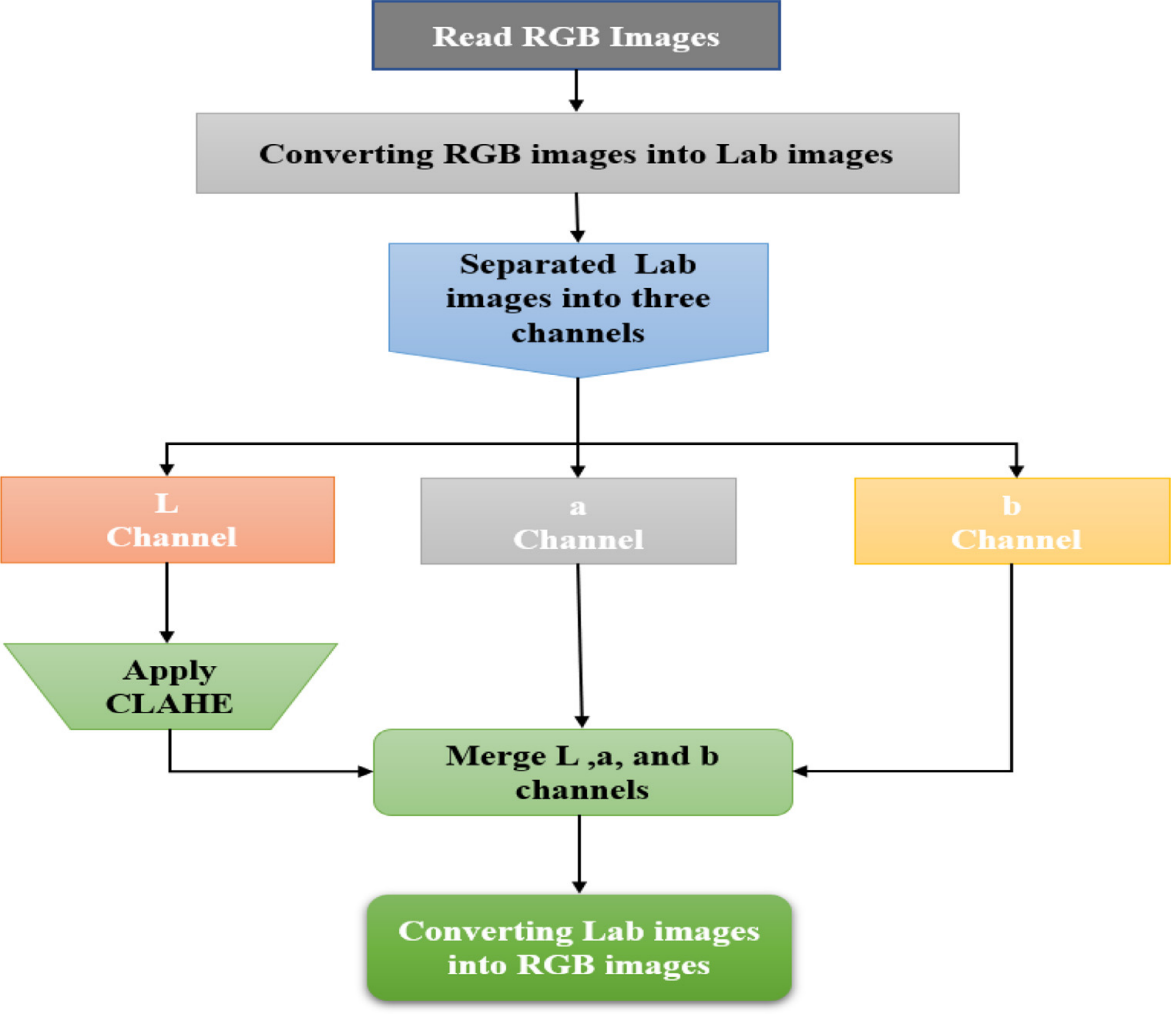
Diagram of the image enhancement process.

Contrast Limited Adaptive Histogram Equalization (CLAHE) is useful for enhancing digital images and is a variant of Adaptive Histogram Equalization (AHE) that supports contrast boosting. CLAHE operates on small regions of the image, called tiles, rather than on the entire image. The neighboring tiles are combined using bilinear interpolation to remove artificial boundaries [4]. Fig. 6 represents the general methodology of the quality improvement process of the dataset.

The process of enhancing our images is as follows:

– **Step 1**: Process of images reading in RGB format.
– **Step 2**: Process of RGB images convertion into Lab images.

In this step, the image has initially used the conversion of RGB color images to Lab color images. The Lab color space uses the concept of coloring based on luminance using the brightness (L) of white-black and chromatic components (a. red-green and b. yellow-blue). The Lab color space is a development of the CIEXYZ color space, with values L that has a value between 0 and 100 that represent respectively black to white, as for the chromatic representation of red-green and yellow-blue colors, each is between -128 and 128 [5].

The conversion from RGB color space to Lab was done using the equation below:

(1) $$L=116\;\times \left(0.299R+0.587G+0.144B^{\frac{1}{3}}\right)-16$$

(2) $$a=500\;\times \left[1.006\;\times {\left(0.607R+0.174G\;+0.201B\right)}^{\frac{1}{3}}-{\left(0.299R+0.587G+0.114B\right)}^{\frac{1}{3}}\right]$$

(3) $$b=200\;\times \left[{\left(0.299R\;+\;0.587G\;+\;0.114B\right)}^{\frac{1}{3}}\;-0.846\;\times {\left(0.066G\;+1.117B\right)}^{{}^{\frac{1}{3}}}\right]$$

**Fig. 7:**
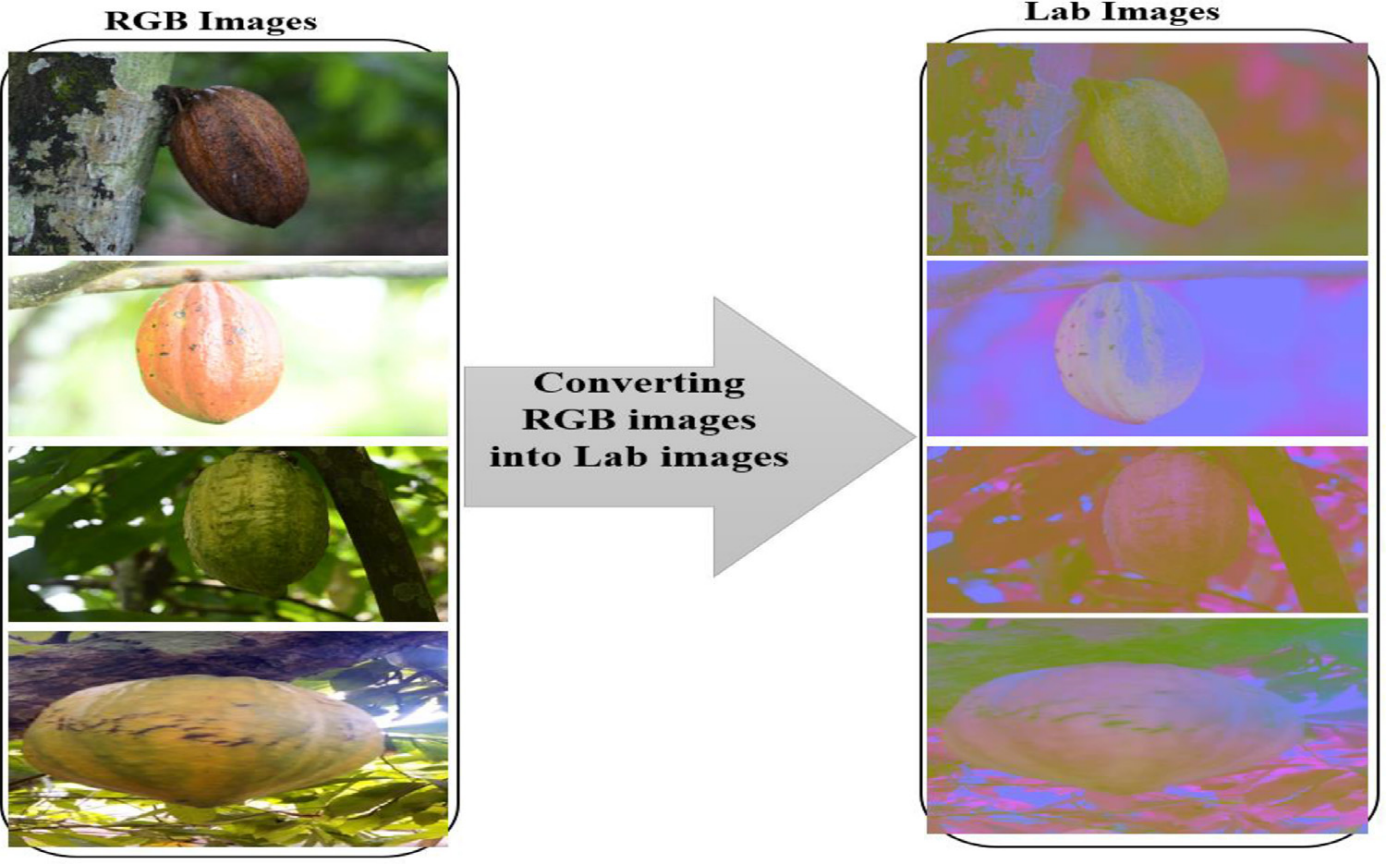
Converting RGB images to Lab images.

Fig. 7 shows the images convertion from RGB color space to Lab

– **Step 3**: Separation of the different channels, in this case, the L, a, and b channels

**Fig. 8:**
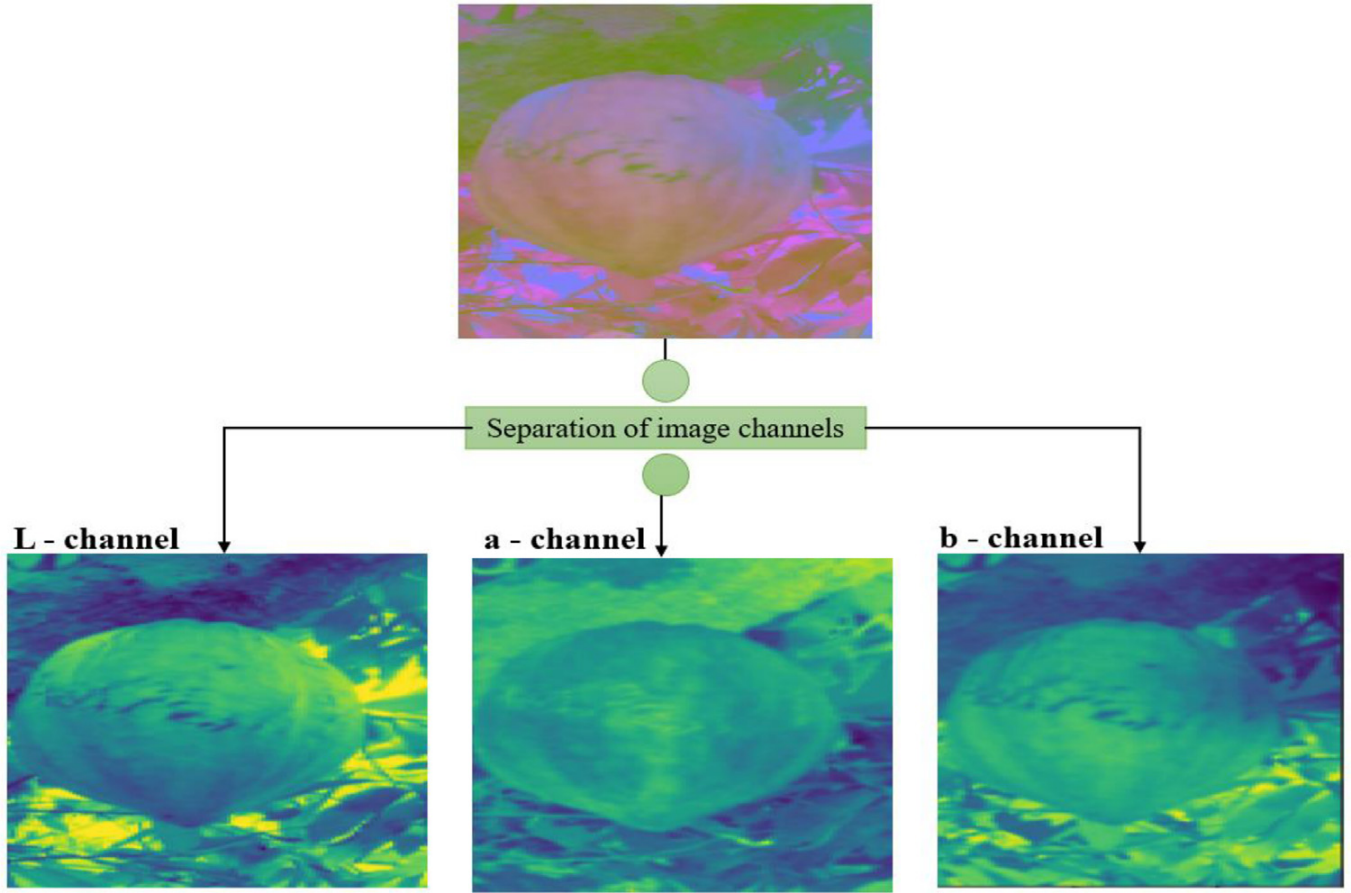
Separation of the different channels of the Lab images.

Fig. 8 *shows the image of the color space separated into three channels*

– **Step 4:** We now applied the CLAHE algorithm which follows these five steps
∘ Divide the image into small regions.
∘ Then use another word on the mapping functions of the local histogram.
∘ Then another word the clipping point of the histogram.
∘ And another expression the function to each region.
∘ Finally, he reduces the noise by the background subtraction method.

**Fig. 9:**
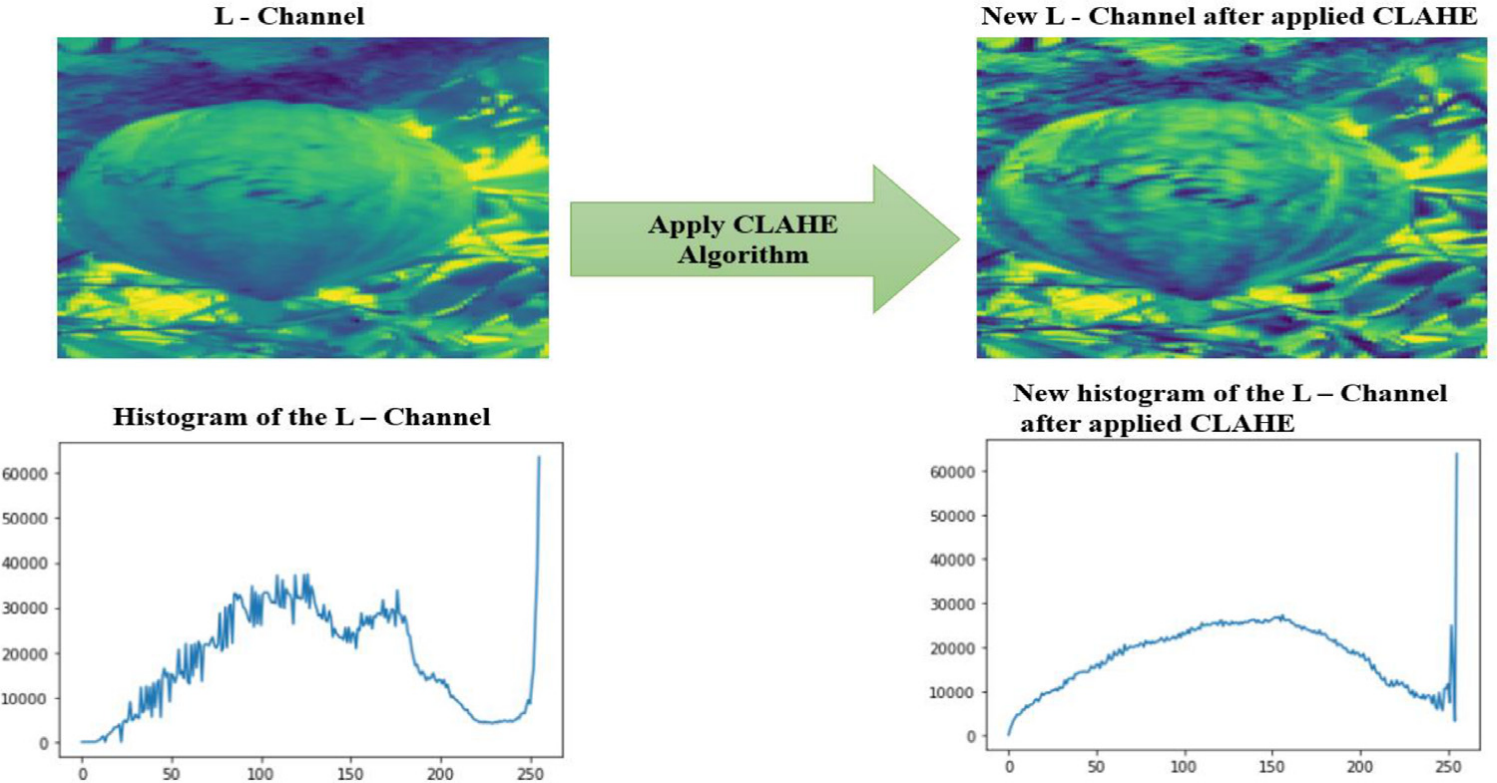
Image and histogram of the L channel before and after application of the CLAHE algorithm.

Fig. 9 shows the result obtained after the application of the CLAHE algorithm on the L channel of the image

– **Step 5:** Fusion of the channels L, a, and b to obtain images in color space Lab was made.

**Fig. 10:**
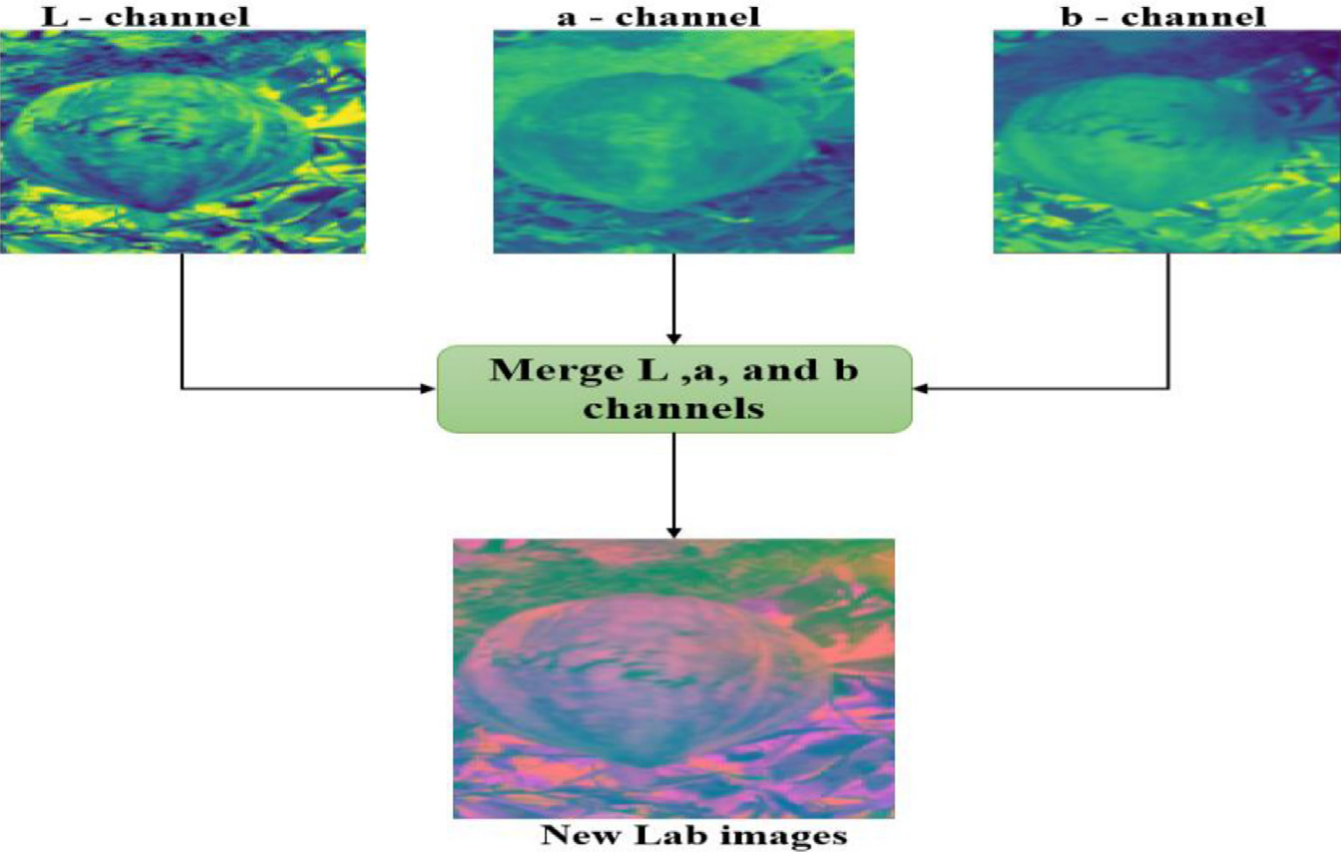
Merge of the L, a and b channels to obtain a new image in Lab color space. (For interpretation of the references to color in this figure legend, the reader is referred to the web version of this article.)

Merge the L, a, and b channels, as shown in Fig. 10

**Fig. 11:**
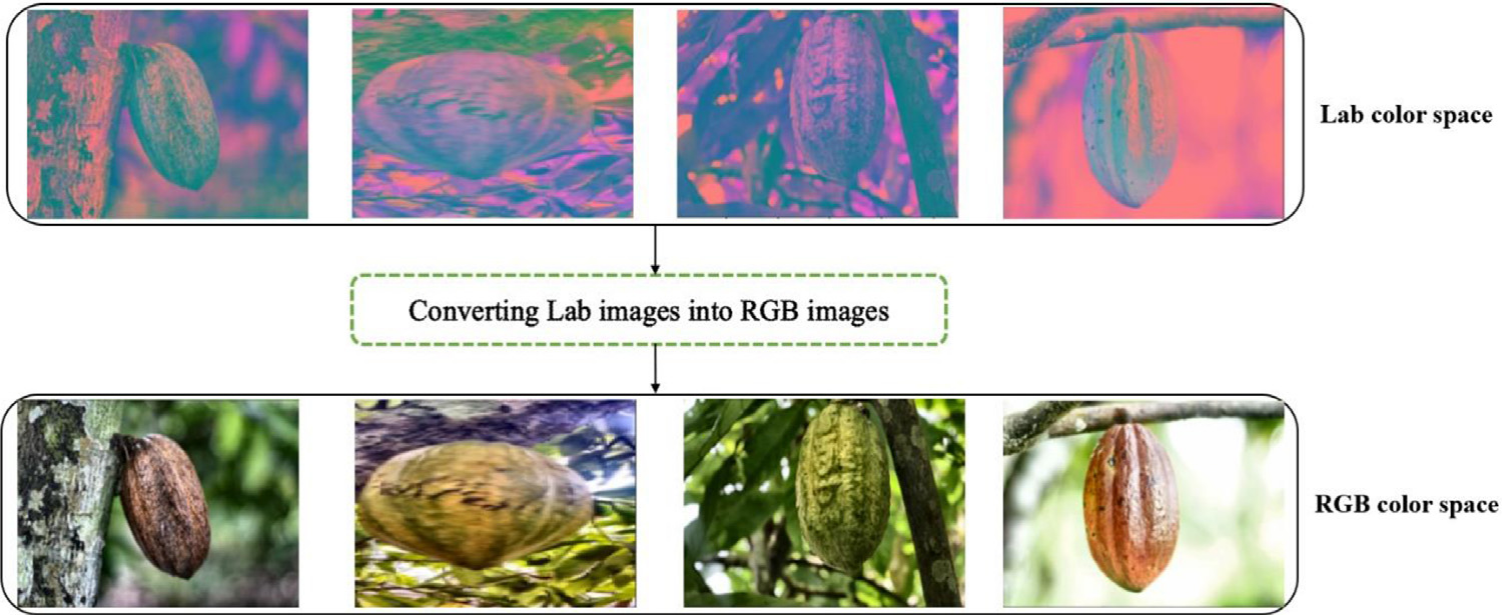
Converted images from Lab color space to RGB color space. (For interpretation of the references to color in this figure legend, the reader is referred to the web version of this article.)
• Labeling

– **Step 6:** Finally, we converted new image generated in the Lab color space to the RGB color space as shown in Fig. 11.

**Fig. 12:**
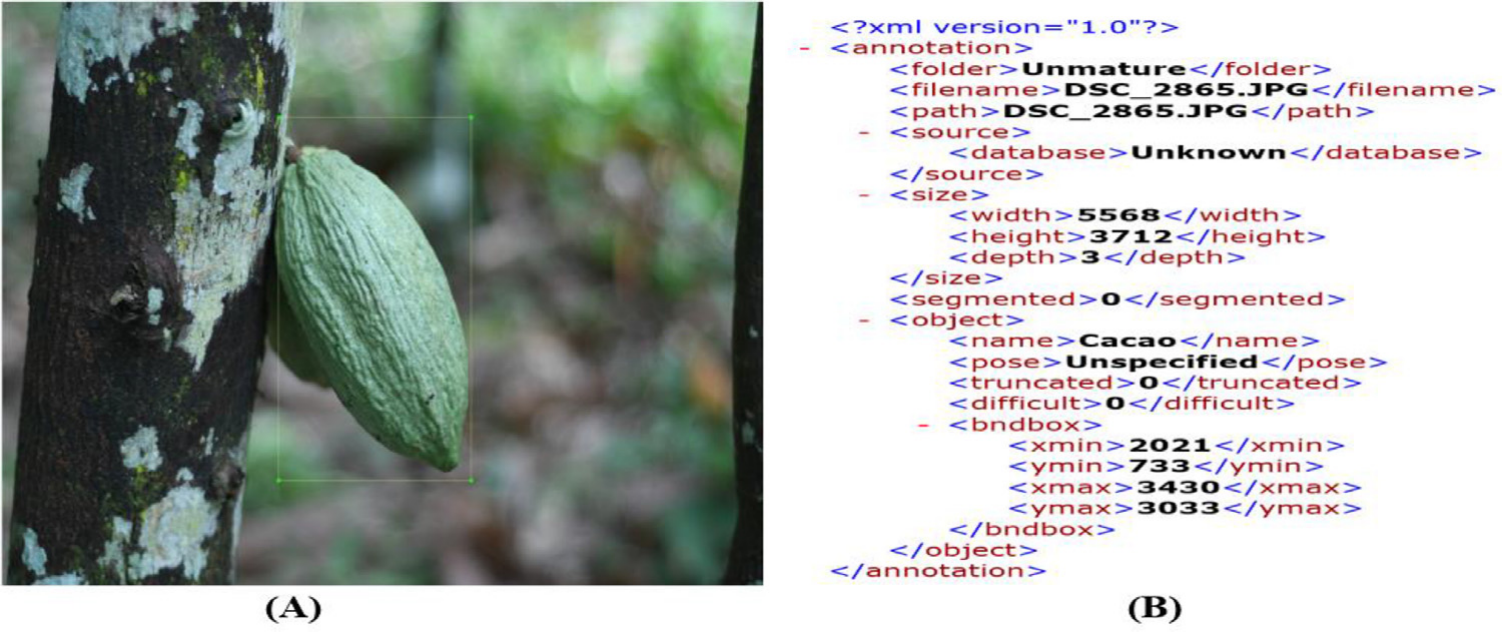
Annotation of cocoa pod. (A) Annotated image. (B) XML document.

Data tagging refers to the process of adding tags or labels to raw data such as images, videos, text, and audio. These tags form a representation of the class of objects to which the data belongs and help a machine learning model learn to identify that particular class of objects when encountered in data without tags. The Labeling tool is used to annotate the positional information of cocoa pods in the image. It is annotated according to the PASCAL VOC 2007 dataset format which represents a dataset containing several labeled images and annotated files. It automatically generates corresponding XML annotation files [6,7]. Fig. 12 shows an image and the file generated.

## Declaration of Competing Interest

The authors declare that they have no known competing financial interests or personal relationships that have, or could be perceived to have, influenced the work reported in this article.

## Data Availability

CocoaMFDB (Original data) (Mendeley Data).
